# Responses to affect subtypes differentially associate with anxious and depressive symptom severity

**DOI:** 10.1371/journal.pone.0235256

**Published:** 2020-07-02

**Authors:** Rebekah J. Mennies, Samantha L. Birk, Julia A. C. Case, Thomas M. Olino

**Affiliations:** Department of Psychology, Temple University, Philadelphia, Pennsylvania, United States of America; The University of Sydney, AUSTRALIA

## Abstract

Responses to affect include cognitive processes (i.e., perseverative vs. non-perseverative) and valence (i.e., modulation of positive vs. negative affect). However, little research has examined how the factor structure of responses to affect is defined along one or both of these dimensions. The present study conducted an exploratory factor analysis (EFA) of items from assessments of repetitive negative thinking, rumination on positive affect (PA), and dampening. We also examined the associations between emergent factors and measures of depressive symptoms, social anxiety symptoms, and non-social state anxiety. EFA results suggested a three-factor model of repetitive negative thinking, dampening, and rumination on PA. There was a significant association between repetitive negative thinking and dampening factors, but not between other factors. Repetitive negative thinking and dampening were associated with greater internalizing symptoms, whereas rumination on PA was associated with fewer internalizing symptoms. These findings clarify the structure of these responses to affect and their differential associations with symptoms, which may be used to tailor cognitive interventions for anxiety and/or depression.

## Introduction

Negative repetitive thoughts are hallmark correlates of depressive and anxiety disorders (e.g., [1]). More recently, there has been interest in studying a repetitive focus on positive affect (PA) known as rumination on PA [2]. However, there have been few examinations of how these and other responses to affect (e.g., dampening, or the focus on negative aspects of a situation to attenuate PA) reflect similar or dissimilar processes. That is, do various repetitive thought dimensions reflect a similar *process* of thinking which is distinct from other non-repetitive responses to affect (such as dampening), are these and dampening distinguished by *valence* rather than process (i.e., amplification of negative vs. positive mood states), or do both distinctions have empirical support? Additionally, the construct of rumination on PA has been studied using thought content to distinguish between emotion-focused and self-focused rumination on PA, while repetitive negative thoughts have not been broken down by content. Importantly, though there are consistent associations between rumination on negative affect (NA) and greater depressive and anxiety symptom severity (e.g., [1, 3, 4]), there are mixed findings reported for rumination on PA and these symptoms (e.g., [2, 5, 6]). Thus, additional work is needed to examine how repetitive negative and positive thinking are associated and implicated in internalizing problems.

As proposed by Nolen-Hoeksema [7], a ruminative response style to NA or negative events may sustain NA and potentially culminate in depressed mood. Other responses to NA are also characterized by recursive focus, such as worry and post-event processing (PEP). Accordingly, perseverative engagement in intrusive negative cognitions about events, or repetitive negative thinking (RNT), may similarly amplify NA. In contrast, rumination on PA, or the recursive focus on positive attributes, mood, or events, enhances the experience of PA [8, 9]. As such, RNT and rumination on PA are similar in their repetitive focus (i.e., cognitive process), yet RNT involves perseveration with negative valence while rumination on PA is defined by perseveration with positive valence. A dampening response to affect is not characterized by repetitive focus, but instead consists of focusing on negative aspects of a situation in a manner which attenuates PA (i.e., shifting attention away from PA rather than hyper-focusing on PA or NA). Like RNT, dampening involves greater attention towards negative rather than positive aspects of mood or events; and like rumination on PA, dampening is defined by the modulation of positive affect, but unlike rumination on PA, dampening attenuates rather than amplifies PA. Thus, these responses to affect are defined by both shared and distinct elements across dimensions of cognitive process (i.e., perseverative or non-perseverative) and valence (modulation of NA or PA). However, it remains unclear whether these proposed responses to affect reflect shared or distinct latent dimensions based on cognitive process and/or the affect valence.

As an initial step towards addressing this question, the psychometric properties of assessments of RNT, rumination on PA, and dampening have been assessed, either individually or with only rumination on PA and dampening items included in factor analysis. In particular, worry is highly correlated with rumination [10, 11], and both worry and PEP involve perseverative focus on negative aspects of an event. Thus far, research has consistently found that RNT is a single-factor construct when assessed using the brief version of the Repetitive Thinking Questionnaire (RTQ), which includes items that assess PEP, worry, and rumination [12, 13]. The Responses to Positive Affect (RPA) questionnaire is a commonly used measure of rumination on PA and dampening [2]. Examination of the factor structure of the RPA questionnaire has typically supported a three-factor model of (1) emotion-focused rumination on PA, defined by focusing on positive emotions, (2) self-focused rumination on PA, defined by focusing on the positive meaning of events for one’s confidence and sense of self, and (3) dampening, defined by focusing on negative aspects of a situation and a shift in mood which reduces PA [2, 9, 14]. Emotion-focused and self-focused rumination on PA tend to be strongly correlated (*r* = .44-.90; [5, 8]). As a result, some researchers have argued that these dimensions reflect a single construct [8, 15], and the utility of this distinction is unclear. Taken together, these study findings indicate that although frequently assessed within the same measure, dampening and rumination on PA are distinct constructs, and rumination on PA may be more parsimoniously captured by one factor.

Consistent associations have been reported between rumination on NA and dampening (e.g., [2, 14, 15]). However, preliminary work on the relationships between rumination on NA and rumination on PA has yielded mixed results. Some studies have found that emotion-focused—but not self-focused—rumination on PA is associated with greater rumination on NA [2, 16]. Conversely, another study found that a composite score of self- and emotion-focused rumination on PA was associated with reduced trait-level—but not state-level—rumination on NA [15]. Further still, another study found no significant associations between rumination on PA and rumination on NA [14]. Finally, studies have also reported mixed results with regard to associations between rumination on PA and dampening. For example, Li et al. [15] found that rumination on PA and dampening were negatively associated at trait level, but positively associated at state level. However, other studies have found non-significant associations between these constructs [5, 17].

Research has also investigated the link between these responses to affect and dimensions of psychopathology. Rumination on NA is robustly associated with greater anxiety and depressive symptom severity [1, 4] and depressive episode onset and maintenance [18, 19, 20]. RNT also has been linked to greater anxiety and depressive symptom severity [5, 10, 12, 13, 21].

Rumination on PA is theorized to contribute to mania, with research indicating greater levels of rumination on PA among individuals with bipolar disorder than control participants [16, 22]. Associations between rumination on PA and internalizing symptoms are less frequently examined and results of those studies are mixed. For example, greater emotion-focused and self-focused rumination on PA may be protective against depressive symptoms [2, 15, 23, 24, 25], though some studies have found non-significant associations between rumination on PA and depressive symptoms [5, 6, 14, 16, 26]. Further, preliminary research has linked greater emotion-focused rumination on PA to reduced anxiety severity [6, 9]. In contrast, one study examining the association between rumination on PA and multiple dimensions of anxiety symptom subtypes found modest *positive* associations between *self*-focused rumination on PA and panic disorder and agoraphobia symptoms (but no association with other types of anxiety), and no significant associations between emotion-focused rumination on PA and anxiety [26]. With regard to dampening, another response to PA, research has found that it is associated with greater depressive symptom severity [14, 16, 15, 24, 26, 27]–even when controlling for rumination on NA–[2, 28], greater anxiety symptoms [26], and comorbid depression and anxiety symptoms [5, 9, 17].

Though research has examined the factor structure of an assessment of rumination on PA and dampening, as well as the factor structure of RNT, no research has conducted a joint exploratory factor analysis (EFA) across items assessing rumination on PA, dampening, and RNT. Our primary goal is to examine whether responses to affect represent overlapping or distinct constructs along dimensions of process and valence. An exploratory approach will aid in evaluating the validity of these constructs as distinct rather than falling along opposite sides of a shared continuum; this, in turn, will inform future measurement of responses to affect. We expect that analyses will demonstrate one of at least three structures: (1) a two-factor structure with responses that influence particular affect states (i.e., PA and NA regulation), (2) a two-factor structure with responses with shared cognitive process (i.e., perseverative vs. non-perseverative responses to affect), or (3) a three-factor structure separated by both dimensions of affect valence and cognitive process. To evaluate these possibilities, joint analyses of all items is critical. Further, it remains unclear whether rumination on PA will be even further differentiated by emotion-focused and self-focused factors of rumination on PA. Thus, this exploratory approach will inform whether the construct of rumination on PA is most accurately assessed as unitary or multidimensional.

We will also examine the relationships between factors emerging from EFA models with one another and both depression and anxiety using confirmatory factor analysis (CFA). Prior work examining the associations between these responses to affect and internalizing symptoms individually has yielded mixed results. Here, we examine the relationships between responses to affect and various dimensions of internalizing symptoms within a single model. As the literature indicates that the relationship between PA and internalizing symptoms may operate differently across internalizing dimensions (i.e., depression vs. social anxiety vs. other forms of anxiety; [29, 30, 31, 32, 33, 34]), we add to existing work by modeling associations between emergent factors and (1) depressive symptoms, (2) social anxiety symptoms, and (3) non-social state anxiety symptoms simultaneously. Thus, results will provide information about the dimensions along which responses to affect fall psychometrically, inform whether rumination on PA is best assessed as unitary or multidimensional, and further elucidate the relationship between these responses to affect—and their relationship with various internalizing symptom dimensions—within a single model.

## Method

### Participants

Participants were 198 (139 female) undergraduate students ranging in age from 18 to 45 years of age (*M*_*age*_ = 21.41 years, *SD* = 3.74). One-hundred-and-two (51.5%) participants reported their race as Caucasian, 44 (22.2%) as African American, 31 (15.7%) as Asian, eight (4.0%) as more than one race, and eight (4.0%) as “Other”. In addition, five participants (2.5%) did not report their race. In regards to ethnicity, the sample was largely non-Hispanic (73.2%).

### Measures

#### Responses to positive affect

Participants completed the Responses to Positive Affect Questionnaire (RPA; [2]). The RPA questionnaire is a 17-item self-report questionnaire that assesses frequency of different responses to PA beginning with the general prompt of, “When you are feeling happy, how often do you…” followed by different responses. In the initial validation, three subscales were found: self-focused rumination on PA (4 items; e.g., “Think I am achieving everything”), emotion-focused rumination on PA (5 items; e.g., “Think about how happy you feel”), and dampening of PA (8 items; e.g., “Remind yourself these feelings won’t last”). Responses are rated on a 4-point scale from one (*almost never*) to four (*almost always*). In previous studies, the RPA questionnaire has demonstrated internal consistency [2, 5]. The RPA questionnaire demonstrated good internal consistency in the current sample as well (*α* = .87).

#### Repetitive negative thinking questionnaire

Participants’ levels of RNT were assessed with the brief Repetitive Thinking Questionnaire (RTQ-10; [12, 13]. The RTQ-10 is a 10-item self-report questionnaire assessing trait RNT (e.g. “I have thoughts or images about all my shortcomings, failings, faults, and mistakes”) in response to feeling “distressed or upset.” Items in the RTQ-10 were derived from existing measures of RNT, including the Penn State Worry Questionnaire (PSWQ; [35]), the Ruminative Responses Scale (RRS; [7]), and the Post-Event Processing Questionnaire-Revised (PEPQ-R; [36]). The RTQ-10 is a brief version of the 27-item RNT scale of the Repetitive Thinking Questionnaire, consisting of the ten items that loaded most strongly onto the RNT factor of the full-length measure; the RTQ-10 correlated highly with the longer 27-item RNT scale [12]. Responses are rated on a 5-point scale from one (*not at all true)* to five (*very true*). In previous studies, the RTQ-10 has demonstrated strong internal consistency (all αs > .89) in clinical [13, 37] and non-clinical [12, 13] samples. The RTQ-10 demonstrated excellent internal consistency in the current sample as well (*α* = .96).

#### State anxiety

Participants’ anxiety was assessed using the state items from the State Trait Anxiety Inventory (STAI; [38]). The STAI is a 20-item self-report assessment of anxiety at the current moment (e.g. “I feel calm”). Responses are rated on a 4-point scale from one (*not at all*) to four (*very much so*). In previous studies, the STAI has shown internal consistency (all αs > .90; [38, 39]. The STAI demonstrated adequate internal consistency in the current sample as well (*α* = .92). The STAI was z-scored to be consistent with other outcome variables of interest.

#### Social anxiety symptoms

Participants’ levels of social anxiety symptoms were assessed using the Liebowitz Social Anxiety Scale–Self-Report (LSAS-SR; [40]) and the Social Phobia Scale (SPS; [41]). The LSAS-SR is a 24-item self-report questionnaire that measures the severity of anxiety in social interaction (11 items, e.g. “Talking to someone in authority”) and performance situations (13 items, e.g. “Writing while being observed”). Ratings of fear and avoidance are completed on a 4-point Likert scale from zero (*none* and *never*) to three (*severe* and *usually*). In previous studies, the self-report version of the LSAS has shown adequate internal consistency (all αs > .79) and compares well to the clinician-administered version [40, 42]. The SPS is a 20-item self-report questionnaire that assess social anxiety symptoms (e.g., “I have become anxious if I have to write in front of people”). Responses are rated 5-point scale from 0 (*not at all characteristic or true or me*) to 4 (*extremely characteristic or true of me*). In previous studies, the SPS has shown good internal consistency (all αs > .87; [43, 44]. Both the LSAS and SPS demonstrated excellent internal consistency in the current sample (αs = .95 and .95, respectively). The association between the LSAS and SPS was strong (*r* = .75, *p* < .001). Thus, a summary social anxiety composite was computed by averaging the z-scores of the total scores of these measures.

#### Depressive symptoms

Depression symptoms were assessed using the Center for Epidemiological Studies Depression Scale (CESD; [45]) and the Patient-Reported Outcomes Measurement Information System Depression Scale (PROMIS-D; [46, 47]). The CESD is a 20-item self-report questionnaire that assesses the frequency of current depressive thoughts and behaviors in the past week (e.g. “I was bothered by things that usually don’t bother me”) on a 4-point scale from zero (*rarely or none of the time*) to three (*most or all of the time*). The PROMIS-D is an 8-item self-report questionnaire that assesses depressive symptoms in the past week, e.g. “I felt worthless,” on a 5-point scale from 1 (*never*) to 5 (*always*). Both the CESD [45] and the PROMIS-D [47] have demonstrated good internal consistency in previous research (αs > .85 and .83, respectively). The internal consistency for the CESD and PROMIS-D were adequate and excellent in the current sample (αs = .78 and .95, respectively). The association between the CESD and PROMIS-D was strong (*r* = .77, *p* < .001). Thus, a summary depression composite was computed by averaging the z-scores of the total scores of these measures.

### Procedure

Undergraduate students at Temple University requested appointments using an online scheduling system. Upon arrival at the laboratory, procedures were reviewed with participants, and all participants provided informed consent. Participants received two and a half credits towards a class requirement for their participation, which included a battery of behavioral tasks and self-report measures. An Institutional Review Board at Temple University approved all study procedures.

### Data analysis

We used all items from the RTQ-10 and the RPA questionnaire in our EFA models. First, we conducted an EFA model with an oblique oblimin rotation to permit associations between emergent factors. Second, we utilized structural equation modeling (SEM) to examine the associations between the emergent factors and internalizing symptoms (measured by the depression and social anxiety composites and the state anxiety score).

Analyses were performed using Mplus 7.31 software [48] and the psych package [49] in R. Because the distribution of responses for several of the RNT and RPA individual items were skewed, response options were treated as ordinal and analyses were conducted using the mean and variance adjusted weighted least squares estimator (WLSMV; [50]). Model selection was driven by both statistical criteria and interpretability of model solutions. Statistical criteria included model fit indices (e.g., [51]) from structural equation modeling (SEM), and we present eigenvalues for complete reporting. SEM indices include the comparative fit index (CFI; [52]) and the root mean square error of approximation (RMSEA; [53]), which should be greater than .90 [54] and between .05 and .10 [55], respectively, to suggest good overall model fit. We also conducted a parallel analysis on the polychoric correlation matrix using the fa.parallel function with 1000 replications and the Velicer minimum average parcel (MAP) procedure in the psych package. These indices, along with interpretability of the factors based on factor content and available theory, were used to determine which model to retain.

## Results

Correlations between all scales administered for this report are presented in Table 1. According to the CESD, 64 individuals endorsed clinical levels of depression based on a cut-off value of 21 [56]. In addition, 78 individuals endorsed clinical levels of social anxiety according to the LSAS based on a cut-off value of 60 [57].

**Table 1:** Correlation matrix for self-report measures with mean and standard deviations.

	RTQ	RPA_Emo	RPA_Self	RPA_Damp	STAI	LSAS	SPS	CESD	PROMIS-D
RTQ									
RPA_Emo	.05								
RPA_Self	-.01	.71**							
RPA_Damp	.44**	.09	.12**						
STAI	.40**	-.25**	-.22**	.35**					
LSAS	.47**	-.05	-.10	.49**	.44**				
SPS	.57**	-.04	-.06	.50**	.53**	.75**			
CESD	.55**	-.12	-.14	.48**	.64**	.51**	.61**		
PROMIS-D	.57**	-.02	-.05	.49**	.57**	.48**	.57**	.77**	
Mean	28.15	9.42	6.51	7.22	38.20	53.11	21.53	17.35	14.06
SD	11.75	3.64	3.02	5.58	11.90	24.81	16.35	10.83	7.25
Skewness	.14	-.32	-.01	.79	.34	.27	.62	.73	1.61
Kurtosis	-1.05	-.63	-.54	-.01	-.66	-.60	-.40	.09	2.10

** *p* < .001. RTQ = Repetitive Negative Thinking Questionnaire, Total; RPA_Emo = Responses to Positive Affect Questionnaire, Emotion-focused subscale; RPA_Self = Responses to Positive Affect Questionnaire, Self-focused subscale; RPA_Damp = Responses to Positive Affect Questionnaire, Dampening subscale; LSAS = Liebowitz Social Anxiety Scale, Total; SPS = Social Phobia Scale, Total; STAI = State Trait Anxiety Inventory, Total; CESD = Center for Epidemiological Studies Depression Scale Total; PROMIS-D = Patient-Reported Outcomes Measurement Information System Depression Scale Total

An EFA was then conducted with all items from the RTQ-10 (RNT) and RPA questionnaire (rumination on PA, dampening). The EFA was conducted with an oblique rotation to permit associations between emergent factors. Table 2 provides eigenvalues and model fit information for the one through nine factor solutions. Supplementary material presents the factor loadings for the complete set of solutions. Eigenvalues are presented for completeness, but not relied on to inform model selection (e.g., [51]). Parallel analyses suggested retaining nine factors, and the MAP analysis suggested retaining the two factor solution. These indices provided guidance on the range of factors to be explored. Model fit indices and interpretability of results were also considered to determine the number of factors indicated by the data. Models with fewer number of factors were preferred over those with more, and models with fewer cross-loadings were preferred over models with more cross-loadings. Preferred structure in the present study was defined as each item showing a primary loading of at least .35 of greater (i.e., explained more than at least 12% of the indicator variance) with weaker secondary or cross-loadings (i.e., primary loadings double the magnitude of secondary loadings).

**Table 2:** Exploratory factor analysis: Model fit indices across emergent solutions.

	Observed Eigenvalue	Percent Variance Explained	Cumulative Percent Variance Explained	*χ*^*2*^	*df*	*p*_*χ2*_	*CFI*	*RMSEA*	Fit Relative to Previous Model, *χ*^*2*^	Fit Relative to Previous Model, *df*	Fit Relative to Previous Model, *p*_*χ2*_
1 Factor	9.75	36.10	36.10	3093.41	324	<0.001	.697	.208 (.201-.214)	N/A	N/A	N/A
2 Factors	5.72	21.17	57.28	1411.15	298	<0.001	.878	.137 (.130-.145)	799.33	26	<0.001
3 Factors	3.40	12.60	69.88	683.72	273	<0.001	.955	.087 (.079-.095)	343.84	25	<0.001
4 Factors	1.01	3.74	73.62	513.92	249	<0.001	.971	.073 (.064-.082)	159.77	24	<0.001
5 Factors	0.92	3.40	77.02	390.00	226	<0.001	.982	.061 (.050-.071)	104.73	23	<0.001
6 Factors	0.74	2.74	79.76	285.01	204	<0.001	.991	.045 (.032-.057)	87.66	22	<0.001
7 Factors	0.72	2.66	82.42	233.50	183	<0.01	.994	.037 (.021-.051)	50.84	21	<0.001
8 Factors	0.57	2.10	84.51	189.41	163	>0.05	.997	.029 (.000-.045)	43.91	20	<0.01
9 Factors	0.46	1.72	86.23	163.34	144	>0.05	.998	.026 (.000-.044)	26.99	19	>0.05

The fit of the model with at least three factors demonstrated at least adequate model fit, with excellent fit (CFI>.95) indicated by CFI = .955 and adequate fit (less than .10) indicated by the point estimate of the RMSEA (.087; [55]). Though the RMSEA is modestly higher than is recommended in more recent guidance [58], the upper limit of the RMSEA CI (.095) did not exceed .10, which is frequently cited as an additional marker of fit [59]. The three-factor solution accounted for 69.88% of variance in the data. The three-factor model was a significantly better fit to the data than the two-factor model, which, in turn, fit the data significantly better than the one-factor model. All items uniquely loaded onto one factor each, without any substantial cross-loadings, in the three-factor model at .35 or greater.

Although the models successively fit the data better as the number of factors increased (Table 2), interpretability of these factors became less clear. In the four-factor model solution, no items loaded strongly enough onto the fourth factor in accordance with our threshold of ≥.35. Further, each of these items substantially cross-loaded onto other factors, a trend which persisted as the number of factors increased across solutions.

On balance of overall model fit and parsimony, we retained the three-factor model as the preferred model (see Table 3 for the complete factor loadings). Five emotion-focused and four self-focused rumination on PA items loaded on the first factor, referred to as the RPA factor. All eight dampening items significantly loaded onto the second factor, referred to as the dampening factor. Ten items assessing trait RNT from the RTQ loaded on the third factor, referred to as the RNT factor. In the three-factor model, there was a significant positive association between the RNT and dampening factors only (*r* = 0.38, *p* < 0.05); the RPA factor was not significantly associated with the RNT factor (*r* = 0.03, *p* > 0.05) or dampening (*r* = 0.09, *p* > 0.05). Analyses were repeated using an orthogonal rotation, and the substantive conclusions of these analyses were consistent.

**Table 3:** Exploratory factor analysis: Factor loadings from three-factor solution with an oblique rotation.

Item	Factor 1 (RPA)	Factor 2 (Dampening)	Factor 3 (RNT)
RPA1: ‘notice how you feel full of energy?’	0.83*	-0.25	0.18
RPA2: ‘savor this moment?’	0.83*	-0.22	0.11
RPA3:'think I am getting everything done?’	0.74*	0.12	-0.06
RPA4:‘think about how you feel up for doing everything?’	0.79*	-0.07	0.07
RPA5:'think I am living up to my potential?’	0.72*	0.22	-0.13
RPA6:‘think this is too good to be true?’	0.33	0.57*	0.04
RPA7:‘think about how happy you feel?’	0.83*	0.07	-0.04
RPA8: 'think about how strong you feel?’	0.84*	0.14	-0.08
RPA9: ‘think about things that could go wrong?’	0.00	0.84*	0.03
RPA10:‘remind yourself that these feelings won't last’	-0.05	0.85*	-0.07
RPA11:‘think people will think I am bragging?’	-0.02	0.71*	0.14
RPA12:‘think about how hard it is to concentrate?’	-0.01	0.59*	0.14
RPA13:‘think I am achieving everything?’	0.61*	0.31	-0.15
RPA14: ‘think I don’t deserve this?’	-0.02	0.86*	0.04
RPA15:‘think my streak of luck is going to end soon?’	-0.01	0.88*	0.09
RPA16:‘think about how proud you are of yourself?’	0.71*	-0.04	-0.06
RPA17:‘think about the things that have not gone well for you?’	0.06	0.73*	0.14
RTQ1: ‘I have thoughts or images about all my shortcomings, failings, faults, mistakes’	0.01	0.09	0.79*
RTQ2: ‘I have thoughts or images about events that come into my head even when I do not wish to think about them again’	-0.08	-0.02	0.89*
RTQ3: ‘I have thoughts or images that I won’t be able to do my job	-0.03	0.14	0.77*
RTQ4: ‘I have thoughts or images that are difficult to forget’	-0.01	0.05	0.83*
RTQ5: ‘Once I start thinking about the situation, I can’t stop’	0.07	0.05	0.83*
RTQ6: ‘I notice that I think about the situation’	0.03	-0.05	0.88*
RTQ7: ‘I have thoughts or images of the situation that I try to resist thinking about’	0.01	-0.04	0.95*
RTQ8: ‘I think about the situation all the time’	0.04	0.04	0.86*
RTQ9: ‘I know I shouldn’t think about the situation, but can’t help it’	0.01	0.05	0.89*
RTQ10: 'I have thoughts or images about the situation and wish it would get better’	0.02	0.00	0.88*

*Meets significance threshold of factor loading >.35

**Fig 1:**
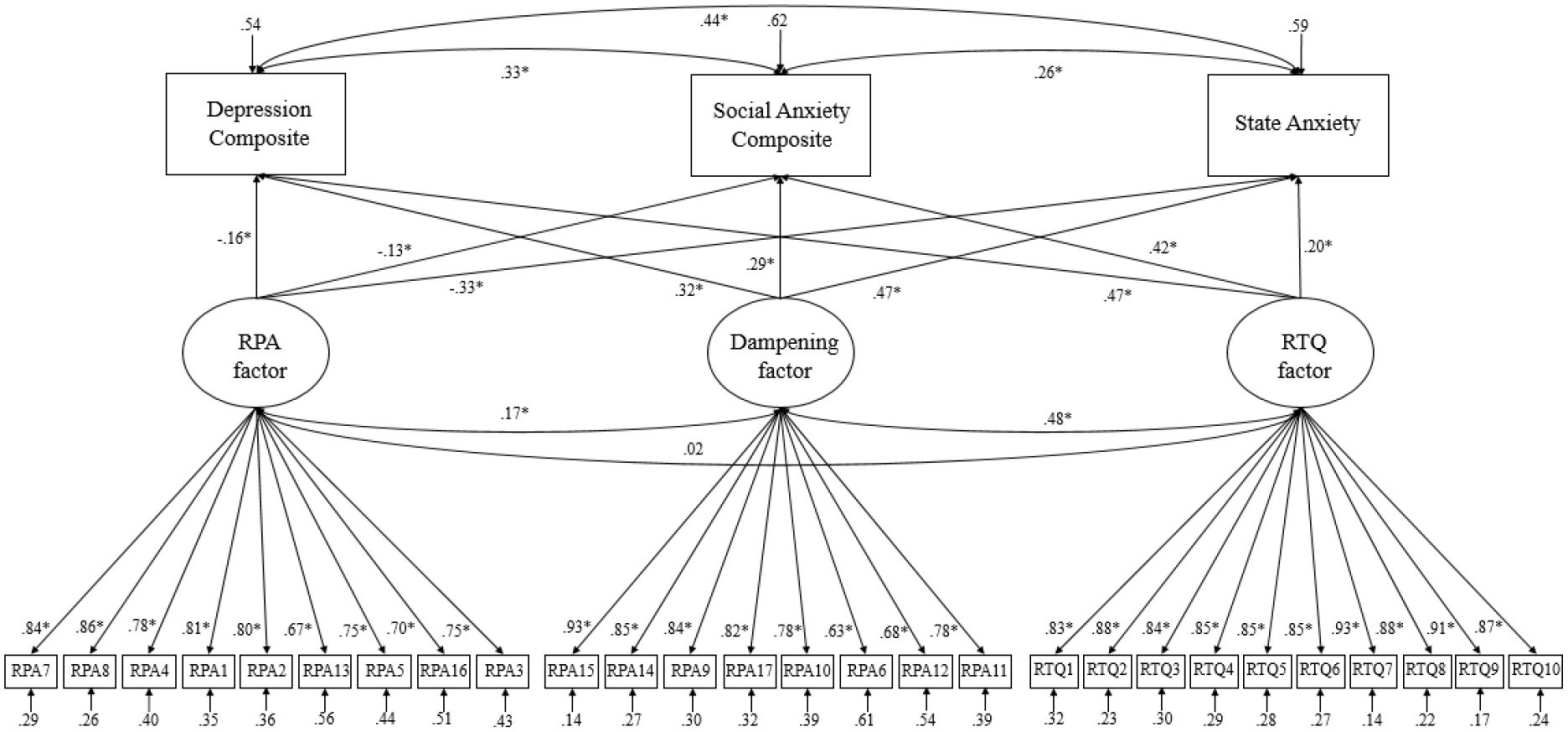
SEM: Model diagram with standardized path estimates.

Next, we examined the associations between the three emergent factors and internalizing symptoms using SEM (Fig 1 provides standardized path estimates). In this model, we specified the measurement of the RPA, dampening, and RNT factors in a confirmatory factor model with the composite depression, composite social anxiety, and non-social state anxiety variables regressed on the latent factors. This model was an adequate fit to the data, χ^2^(393) = 724.85, *p* < 0.001, RMSEA = 0.07 (90% CI: 0.06–0.07), CFI = 0.96. The RNT factor was associated with greater composite depressive symptoms (*b* = .53, SE = .08, *t* = 6.66, *p* < 0.001), composite social anxiety symptoms (*b* = .48, SE = .08 *t* = 5.78, *p* < 0.001), and state anxiety (*b* = .24, SE = .08, *t* = 2.92, *p* = 0.004). Similarly, the dampening factor was positively associated with composite depressive symptoms (*b* = .32, SE = .07, *t* = 4.68, *p* < 0.001), composite social anxiety symptoms (*b* = .29, SE = .07, *t* = 4.21, *p* < 0.001), and state anxiety (*b* = .50, SE = .09, *t* = 5.78, *p* < 0.001). Conversely, the RPA factor was negatively associated with composite depressive symptoms (*b = -*.18, SE = .06, *t* = -3.06, *p* = 0.002), composite social anxiety symptoms (*b* = -.14, SE = .07, *t* = -2.13, *p* = 0.03), and state anxiety (*b* = -.39, SE = .08, *t* = -4.78, *p* < 0.001).

Analyses were repeated without the two RPA items that demonstrated substantial cross loadings on two factors. In these analyses, the final model solution, magnitude of factor loadings, and associations between latent dimensions and dimensions of depression and anxiety were substantively identical. However, the association between the RPA factor and social anxiety was no longer significant (*b* = -.10, *SE* = .06, *t* = -1.63, *p* = .10).

## Discussion

Previous work examined the factor structure of RNT [5, 12, 37] and rumination on PA [8, 15] individually. However, joint analyses have the potential to identify whether there is substantive overlap between these processes. Moreover, though there are consistent associations between RNT and depression and anxiety [1, 3, 4, 11], there are mixed findings for the relationship between rumination on PA and depression and anxiety [2, 5, 6, 9, 14, 15, 17, 23, 24, 26]. This study found that a three-factor model consisting of rumination on PA, dampening, and RNT factors described the data with at least adequate model fit (i.e., excellent fit indicated by the CFI and adequate fit indicated by the RMSEA). The RNT factor was positively associated with the dampening factor but not the RPA factor, and the RPA and dampening factors were also not associated with each other. In addition, results found that both the RNT and dampening factors were positively associated with depression, social anxiety, and non-social state anxiety. Conversely, the RPA factor was negatively associated with depression, social anxiety, and non-social state anxiety.

The three-factor model had factors that were distinguished based on valence and process. Previous research has supported RNT as a single factor [5], and some researchers have argued that the RPA questionnaire is a two-factor construct, with emotion-focused and self-focused rumination on PA reflecting a single construct [8, 15]. Here, a three-factor model of RPA and RTQ-10 items that collapsed the self-focused and emotion-focused rumination on PA into one factor appeared to be more parsimonious and theoretically sound. Similar to previous work, dampening was a separate factor. RNT and rumination on PA are differentiated by valence (negative vs. positive, respectively), but share a similar process of repetitive thinking. RNT and dampening share similar valence (whereby they both enhance negative affect), while differing in process (perseverative vs. non-perseverative). Finally, rumination on PA and dampening also share similar valence (whereby they both alter PA), while also differing in perseverative nature. Despite these similarities, only RNT and dampening were significantly associated. Thus, this supports that tendencies to think perseveratively may take multiple forms, with no evidence of a tendency towards or away from co-occurrence in an unselected undergraduate sample. However, given evidence that individuals with bipolar spectrum disorder experience greater rumination on PA and NA relative to controls [16, 22], while individuals with unipolar depression experience greater rumination on NA only relative to controls [16], risk for or presence of mania may be an important moderator of the association between repetitive negative and positive thinking in future work. Future work should also consider modeling the relationship between responses to affect and manic symptoms, given that rumination on PA is elevated in those with a bipolar spectrum disorder [16, 22].

Our finding that RNT and dampening are positively associated with internalizing symptoms is consistent with previous research finding that RNT [1, 4, 5, 10, 12, 21] and dampening [5, 14, 15, 16, 17, 24, 27, 28] are associated with greater anxiety and depressive symptoms. The current findings show that rumination on PA is negatively associated with depression, social anxiety, and non-social state anxiety. This is consistent with some previous research showing that rumination in response to PA may be protective against depressive symptoms [2, 14, 17, 15, 23, 24]. Further, this finding may aid in understanding the relationship between rumination on PA and internalizing symptoms, as previously mixed findings in the literature may be attributable, in part, to differentiation of self-focused and emotion-focused rumination on PA [2, 5, 6, 9, 14, 15, 17, 23, 24, 26].

The present work contributes to the literature regarding the factor structures of rumination on PA, RNT, and dampening, as well as the associations between these constructs with both depression and anxiety. Rather than focusing solely on rumination on NA, the current study utilized a measure that captures the broader construct of RNT. The current results supported the assessment of RNT by the RTQ-10, which may ease participant burden and be a more parsimonious solution to administering various RNT measures. In addition, although the RPA questionnaire is often considered to have three factors, the current results supported a two-factor solution of rumination on PA and dampening. However, this solution came in the context of the RTQ-10, which may have impacted the RPA structure. These findings are important both in how we assess repetitive thinking and in how we understand the role of repetitive thinking in internalizing symptoms. Specifically, valence appears to impact whether these processes are protective against or contribute to symptoms of depression and anxiety.

The current study has a number of important limitations to note. First, these data come from an undergraduate sample, which may limit the generalizability of the results. Therefore, future work should compare student versus non-student samples, clinical versus non-clinical samples, and adult versus youth and adolescent samples. However, it is important to note that within the current sample, a large minority of participants exceeded clinical cutoffs for depression and social anxiety. Thus, our sample has substantial diversity in levels of symptomatology. Second, the current study utilized the same sample to examine both the validity and utility of the proposed three-factor model. It will be important for future work to validate this model in a separate sample. Third, the data were cross-sectional, which does not provide evidence about the directionality of associations. Fourth, the current study relied solely on self-report measures. Future research is necessary to identify how behavioral, physiological, and neuroimaging data may be utilized to inform a multilevel structure of responses to affect. Fifth, a priori power for EFA is underdeveloped. We had fewer than 10 participants per item analyzed in our model. Thus, results should be interpreted with caution and warrant further attention in larger and more diverse samples. However, our model solution was quite clear with the vast majority of items having primary loadings that were substantially larger than secondary (and tertiary) loadings. This suggests that the factor solution was quite clear. Sixth, measurement differences between the two measures included in the EFA, such as response scales of different magnitudes, differing anchors, and item presentation style (i.e., responding to independent statements vs. questions following an incomplete sentence subject) may have influenced the factor structure. Finally, while we assessed depression, social anxiety, and state anxiety, we did not include an assessment of general trait anxiety, as data on non-social trait anxiety were not collected as part of the larger study. However, variance in state anxiety is attributable to a combination of state, trait, and situation-related anxiety, with test-retest reliabilities ranging from .34 to .96 in prior studies (*M* = .70; [60]). Thus, there is some degree of trait consistency captured by the state measure. This will be important for informing future assessment of these constructs and interventions that target these constructs specifically to reduce symptom severity and improve associated outcomes.

In sum, the present study advances our understanding of RNT, rumination on PA, and dampening as unique constructs which are psychometrically distinguished on dimensions of cognitive process and valence, as well as their relationship with several types of internalizing symptoms. Researchers may also consider developing and validating a single measure which assesses all three constructs simultaneously using a common set of instructions, wording, and Likert scale. Further, future studies may wish to assess rumination on PA as a unitary construct rather than differentiating into emotion-focused and self-focused subtypes. Of additional note, given theoretical and empirical associations between rumination on PA and mania, extensions of the present study should investigate the factor structure of these constructs in clinical samples of individuals with unipolar depressive and bipolar spectrum disorders.

## Supporting information

S1 Table Exploratory factor analysis: Factor loadings from one-factor solution with an oblique rotation. (DOCX)

S2 Table Exploratory factor analysis: Factor loadings from two-factor solution with an oblique rotation. (DOCX)

S3 Table Exploratory factor analysis: Factor loadings from four-factor solution with an oblique rotation. (DOCX)

S4 Table Exploratory factor analysis: Factor loadings from five-factor solution with an oblique rotation. (DOCX)

S5 Table Exploratory factor analysis: Factor loadings from six-factor solution with an oblique rotation. (DOCX)

S6 Table Exploratory factor analysis: Factor loadings from seven-factor solution with an oblique rotation. (DOCX)

S7 Table Exploratory factor analysis: Factor loadings from eight-factor solution with an oblique rotation. (DOCX)

S8 Table Exploratory Factor analysis: Factor loadings from nine-factor solution with an oblique rotation. (DOCX)
