# Supplementary material for: Responses to affect subtypes differentially associate with anxious and depressive symptom severity
Source: PLoS One. 2020 Jul 2;15(7):e0235256. doi: 10.1371/journal.pone.0235256 (PMC7332051; doi:10.1371/journal.pone.0235256)
Supplement: S1 Table — (DOCX) [file pone.0235256.s001.docx]

Supplementary Table 1. *Exploratory Factor Analysis:* *Factor loadings from one-factor solution with an oblique rotation.*

| Item | Factor 1 |
| --- | --- |
| RPA1: ‘notice how you feel full of energy?’ | 0.49 |
| RPA2: ‘savor this moment?’ | 0.47 |
| RPA3:'think I am getting everything done?’ | 0.43 |
| RPA4:‘think about how you feel up for doing everything?’ | 0.46 |
| RPA5:'think I am living up to my potential?’ | 0.42 |
| RPA6:‘think this is too good to be true?’ | 0.51 |
| RPA7:‘think about how happy you feel?’ | 0.58 |
| RPA8: 'think about how strong you feel?’ | 0.57 |
| RPA9: ‘think about things that could go wrong?’ | 0.65 |
| RPA10:‘remind yourself that these feelings won't last’ | 0.63 |
| RPA11:‘think people will think I am bragging?’ | 0.61 |
| RPA12:‘think about how hard it is to concentrate?’ | 0.51 |
| RPA13:‘think I am achieving everything?’ | 0.37 |
| RPA14: ‘think I don’t deserve this?’ | 0.69 |
| RPA15:‘think my streak of luck is going to end soon?’ | 0.79 |
| RPA16:‘think about how proud you are of yourself?’ | 0.35 |
| RPA17:‘think about the things that have not gone well for you?’ | 0.66 |
| RTQ1: ‘I have thoughts or images about all my shortcomings, failings, faults, mistakes’ | 0.78 |
| RTQ2: ‘I have thoughts or images about events that come into my head even when I do not wish to think about them again’ | 0.83 |
| RTQ3: ‘I have thoughts or images that I won’t be able to do my job | 0.77 |
| RTQ4: ‘I have thoughts or images that are difficult to forget’ | 0.79 |
| RTQ5: ‘Once I start thinking about the situation, I can’t stop’ | 0.81 |
| RTQ6: ‘I notice that I think about the situation’ | 0.81 |
| RTQ7: ‘I have thoughts or images of the situation that I try to resist thinking about’ | 0.91 |
| RTQ8: ‘I think about the situation all the time’ | 0.84 |
| RTQ9: ‘I know I shouldn’t think about the situation, but can’t help it’ | 0.88 |
| RTQ10: 'I have thoughts or images about the situation and wish it would get better’ | 0.84 |
