# Supplementary material for: Responses to affect subtypes differentially associate with anxious and depressive symptom severity
Source: PLoS One. 2020 Jul 2;15(7):e0235256. doi: 10.1371/journal.pone.0235256 (PMC7332051; doi:10.1371/journal.pone.0235256)
Supplement: S2 Table — (DOCX) [file pone.0235256.s002.docx]

Supplementary Table 2. *Exploratory Factor Analysis:* *Factor loadings from two-factor solution with an oblique rotation.*

| Item | Factor 1 | Factor 2 |
| --- | --- | --- |
| RPA1: ‘notice how you feel full of energy?’ | 0.77 | -0.07 |
| RPA2: ‘savor this moment?’ | 0.78 | -0.11 |
| RPA3:'think I am getting everything done?’ | 0.74 | -0.02 |
| RPA4:‘think about how you feel up for doing everything?’ | 0.75 | -0.03 |
| RPA5:'think I am living up to my potential?’ | 0.74 | -0.02 |
| RPA6:‘think this is too good to be true?’ | 0.47 | 0.39 |
| RPA7:‘think about how happy you feel?’ | 0.83 | -0.03 |
| RPA8: 'think about how strong you feel?’ | 0.85 | -0.03 |
| RPA9: ‘think about things that could go wrong?’ | 0.35 | 0.62 |
| RPA10:‘remind yourself that these feelings won't last’ | 0.33 | 0.6 |
| RPA11:‘think people will think I am bragging?’ | 0.26 | 0.61 |
| RPA12:‘think about how hard it is to concentrate?’ | 0.2 | 0.52 |
| RPA13:‘think I am achieving everything?’ | 0.66 | 0.03 |
| RPA14: ‘think I don’t deserve this?’ | 0.36 | 0.65 |
| RPA15:‘think my streak of luck is going to end soon?’ | 0.41 | 0.73 |
| RPA16:‘think about how proud you are of yourself?’ | 0.67 | -0.12 |
| RPA17:‘think about the things that have not gone well for you?’ | 0.35 | 0.62 |
| RTQ1: ‘I have thoughts or images about all my shortcomings, failings, faults, mistakes’ | -0.07 | 0.81 |
| RTQ2: ‘I have thoughts or images about events that come into my head even when I do not wish to think about them again’ | -0.18 | 0.86 |
| RTQ3: ‘I have thoughts or images that I won’t be able to do my job | -0.06 | 0.81 |
| RTQ4: ‘I have thoughts or images that are difficult to forget’ | -0.17 | 0.83 |
| RTQ5: ‘Once I start thinking about the situation, I can’t stop’ | -0.03 | 0.83 |
| RTQ6: ‘I notice that I think about the situation’ | -0.09 | 0.84 |
| RTQ7: ‘I have thoughts or images of the situation that I try to resist thinking about’ | -0.12 | 0.92 |
| RTQ8: ‘I think about the situation all the time’ | -0.06 | 0.87 |
| RTQ9: ‘I know I shouldn’t think about the situation, but can’t help it’ | -0.08 | 0.9 |
| RTQ10: 'I have thoughts or images about the situation and wish it would get better’ | -0.08 | 0.86 |
