# Supplementary material for: Responses to affect subtypes differentially associate with anxious and depressive symptom severity
Source: PLoS One. 2020 Jul 2;15(7):e0235256. doi: 10.1371/journal.pone.0235256 (PMC7332051; doi:10.1371/journal.pone.0235256)
Supplement: S7 Table — (DOCX) [file pone.0235256.s007.docx]

Supplementary Table 7. *Exploratory Factor Analysis:* *Factor loadings from eight-factor solution with an oblique rotation.*

| Item | Factor 1 | Factor 2 | Factor 3 | Factor 4 | Factor 5 | Factor 6 | Factor 7 | Factor 8 |
| --- | --- | --- | --- | --- | --- | --- | --- | --- |
| RPA1: ‘notice how you feel full of energy?’ | 0.63 | -0.17 | 0.01 | 0.26 | 0.06 | 0.15 | -0.06 | 0.22 |
| RPA2: ‘savor this moment?’ | 0.69 | -0.09 | -0.02 | 0.31 | -0.1 | 0.12 | -0.2 | 0.06 |
| RPA3:'think I am getting everything done?’ | 0.76 | 0.12 | 0.01 | -0.05 | 0.25 | -0.09 | 0.06 | -0.17 |
| RPA4:‘think about how you feel up for doing everything?’ | 0.71 | 0.03 | 0.02 | 0.15 | 0.07 | -0.16 | 0.13 | 0.07 |
| RPA5:'think I am living up to my potential?’ | 0.52 | 0.15 | -0.06 | 0.13 | 0.33 | -0.04 | 0.01 | -0.07 |
| RPA6:‘think this is too good to be true?’ | 0.17 | 0.59 | 0.08 | 0.26 | -0.05 | -0.13 | 0.24 | -0.14 |
| RPA7:‘think about how happy you feel?’ | 0.09 | 0 | 0 | 0.91 | -0.05 | 0.1 | -0.06 | -0.08 |
| RPA8: 'think about how strong you feel?’ | 0.05 | 0.07 | -0.04 | 0.82 | 0.13 | -0.1 | 0.09 | 0.06 |
| RPA9: ‘think about things that could go wrong?’ | 0.1 | 0.92 | 0.05 | -0.05 | -0.05 | -0.06 | -0.2 | -0.04 |
| RPA10:‘remind yourself that these feelings won't last’ | 0.02 | 0.88 | -0.09 | -0.04 | -0.05 | 0.03 | -0.12 | 0.03 |
| RPA11:‘think people will think I am bragging?’ | -0.08 | 0.6 | 0.16 | 0.08 | 0.05 | 0.26 | -0.06 | -0.13 |
| RPA12:‘think about how hard it is to concentrate?’ | -0.09 | 0.38 | 0.11 | 0.05 | 0.14 | 0.56 | -0.03 | -0.08 |
| RPA13:‘think I am achieving everything?’ | 0.15 | 0.04 | 0 | 0.1 | 0.85 | 0.05 | -0.02 | 0.02 |
| RPA14: ‘think I don’t deserve this?’ | -0.13 | 0.75 | 0.03 | 0.03 | 0.15 | 0.07 | 0.26 | 0.03 |
| RPA15:‘think my streak of luck is going to end soon?’ | -0.06 | 0.8 | 0 | 0.03 | 0.05 | 0.22 | 0.18 | 0.11 |
| RPA16:‘think about how proud you are of yourself?’ | 0.03 | -0.19 | 0.03 | 0.59 | 0.32 | -0.07 | 0.03 | 0.09 |
| RPA17:‘think about the things that have not gone well for you?’ | -0.01 | 0.7 | 0.16 | -0.02 | 0.16 | -0.04 | 0.1 | 0.04 |
| RTQ1: ‘I have thoughts or images about all my shortcomings, failings, faults, mistakes’ | -0.04 | 0.16 | 0.86 | 0.1 | -0.02 | -0.11 | -0.26 | -0.06 |
| RTQ2: ‘I have thoughts or images about events that come into my head even when I do not wish to think about them again’ | -0.08 | -0.05 | 0.99 | 0.02 | 0.03 | -0.06 | -0.05 | -0.18 |
| RTQ3: ‘I have thoughts or images that I won’t be able to do my job | -0.13 | 0.14 | 0.77 | 0.09 | 0.03 | 0.02 | -0.13 | 0.05 |
| RTQ4: ‘I have thoughts or images that are difficult to forget’ | -0.06 | -0.03 | 0.88 | -0.06 | 0.07 | 0.07 | 0.13 | -0.16 |
| RTQ5: ‘Once I start thinking about the situation, I can’t stop’ | 0.21 | 0.1 | 0.71 | -0.02 | -0.19 | 0.13 | 0.13 | 0.06 |
| RTQ6: ‘I notice that I think about the situation’ | 0.21 | 0.01 | 0.77 | -0.16 | -0.03 | 0.1 | -0.04 | 0.17 |
| RTQ7: ‘I have thoughts or images of the situation that I try to resist thinking about’ | 0.06 | -0.09 | 0.93 | -0.02 | 0 | 0.1 | 0.17 | -0.03 |
| RTQ8: ‘I think about the situation all the time’ | 0.04 | 0.13 | 0.67 | 0.05 | -0.15 | 0 | 0.26 | 0.32 |
| RTQ9: ‘I know I shouldn’t think about the situation, but can’t help it’ | -0.03 | 0.11 | 0.73 | 0.02 | -0.03 | 0.03 | 0.02 | 0.35 |
| RTQ10: 'I have thoughts or images about the situation and wish it would get better’ | -0.02 | 0.03 | 0.76 | -0.05 | 0.1 | 0.04 | -0.06 | 0.32 |
